# Development and Validation of Sixplexed Opsonophagocytic Killing Assay for Serotype-Specific Functional Pneumococcal Antibody Measurement

**DOI:** 10.3390/vaccines14030278

**Published:** 2026-03-21

**Authors:** A-Yeung Jang, Hyun Jung Ji, Yu Jung Choi, Eliel Nham, Jin Gu Yoon, Min Joo Choi, Ji Yun Noh, Hee Jin Cheong, Ho Seong Seo, Joon Young Song

**Affiliations:** 1 Division of Infectious Disease, Department of Internal Medicine, Korea University College of Medicine, Seoul 02841, Republic of Korea; grkmcgrid013@kumc.or.kr (A.-Y.J.); pm94@korea.ac.kr (Y.J.C.); e.nham@kumc.or.kr (E.N.); zephirisj9@gmail.com (J.G.Y.); cowgow@naver.com (M.J.C.); jynoh@korea.ac.kr (J.Y.N.); heejinmd@korea.ac.kr (H.J.C.); 2 Vaccine Innovation Center-KU Medicine (VIC-K), Seoul 02841, Republic of Korea; 3 Research Division for Cyclotron Application, Korea Atomic Energy Research Institute, Jeongeup 56212, Republic of Korea; hyunjung@kaeri.re.kr; 4 Department of Radiation Science, University of Science and Technology, Daejeon 34113, Republic of Korea

**Keywords:** *Streptococcus pneumoniae*, pneumococcal vaccine, opsonophagocytic killing assay, serotype

## Abstract

**Background**: Although pneumococcal conjugate vaccines (PCVs) have substantially reduced invasive pneumococcal disease, the emergence of non-vaccine serotypes and antimicrobial-resistant strains has driven the development of higher-valency vaccines. To support functional immune evaluation of these vaccines, we developed and validated a sixplexed opsonophagocytic killing assay (OPA) covering 24 pneumococcal serotypes. **Methods**: Eight additional serotypes, beyond the 16 included in the conventional fourplex OPA, were generated through stepwise natural mutation under increasing concentrations of ciprofloxacin or doxycycline. Assay conditions were optimized by evaluating multiple effector-to-target (E:T) ratios and baby rabbit complement (BRC) concentrations to minimize non-specific killing (NSK). Validation assessed (1) specificity using inhibition OPA with homologous and heterologous polysaccharides, (2) accuracy by comparison with the single-serotype OPA (SOPA), and (3) precision across five independent experiments using the coefficient of variation (CV). **Results**: An E:T ratio of 200:1 combined with 10% BRC consistently maintained NSK below 30% across all assay sets. High serotype specificity was demonstrated by near-complete inhibition following homologous polysaccharide adsorption for all serotypes except serotypes 4 and 8, which exhibited very low opsonic indices. Results from the sixplexed OPA showed strong concordance with SOPA, and overall assay precision was acceptable, with CVs generally below 30% when serotypes with very low opsonic activity were excluded. **Conclusions**: The sixplexed OPA expands functional antibody assessment from 16 to 24 serotypes within four assay sets, providing an efficient and scalable platform for immunogenicity evaluation of current and next-generation high-valency pneumococcal vaccines.

## 1. Introduction

*Streptococcus pneumoniae* (pneumococcus) is a Gram-positive bacterium that commonly colonizes the upper respiratory tract. It is a major human pathogen responsible for both noninvasive and invasive diseases, including pneumonia, meningitis, septicemia, acute otitis media, and sinusitis, particularly in children under 5 years of age and adults aged ≥65 years [1]. More than 100 pneumococcal serotypes have been identified based on the antigenic diversity of their capsular polysaccharides [2,3].

Pneumococcal capsular polysaccharides, the primary virulence factor of *Streptococcus pneumoniae*, have been the main targets of vaccine development, leading to the introduction of pneumococcal polysaccharide vaccines (PPSVs) and pneumococcal conjugate vaccines (PCVs) [4]. Following the introduction of PCV7 in 2007, the incidence of invasive pneumococcal disease (IPD) declined by approximately 90% among children under 5 years of age in the United States [5]. Subsequent replacement of PCV7 with PCV13 further reduced IPD incidence; however, this benefit was accompanied by an increase in non-vaccine serotypes, including 8, 9N, 12F, 22F, and 23A, particularly among young children and older adults [6,7]. The growing prevalence of these non-vaccine serotypes raises concerns about immune escape, which may compromise vaccine effectiveness [8,9].

Serotype replacement, together with the emergence of antimicrobial-resistant strains, remains a major challenge following the introduction of pneumococcal conjugate vaccines [10]. Changes in serotype distribution and resistance patterns vary by geographic region and age group and are strongly serotype-dependent [11,12]. Although PCV implementation has reduced disease caused by antibiotic-resistant vaccine serotypes, antimicrobial resistance among non-vaccine serotypes has become an increasing concern [13]. Historically, vaccine serotypes such as 6A, 19A, and 23F exhibited high levels of penicillin resistance [14,15], whereas non-PCV13 serotypes including 12F, 22F, and 23A have recently been associated with antimicrobial resistance and invasive disease [16]. Notably, several of these serotypes are now included in higher-valency vaccines, reflecting their growing clinical relevance [17]. These trends highlight the need for continued surveillance and the development of vaccines targeting a broader range of serotypes to effectively address emerging antimicrobial resistance and sustain protection against invasive pneumococcal disease [4].

The increasing burden of IPDs caused by non-vaccine serotypes, together with the rising prevalence of multidrug-resistant strains, underscores the need for PCVs with broader serotype coverage, including 20-valent and 24-valent formulations (Appendix A Table A1) [4]. Comprehensive evaluation of functional antibody responses to these expanded serotypes requires high-throughput and scalable immunoassays. In this study, we aimed to develop and validate a sixplexed opsonophagocytic killing assay (OPA) by extending the established fourplex OPA platform to incorporate two additional antibiotic-resistant serotypes per assay set. The selected serotypes (2, 4, 8, 11A, 12F, 17F, 20, and 22F) correspond to those included in investigational 24-valent pneumococcal conjugate vaccines and represent clinically relevant non-vaccine serotypes that have emerged following widespread PCV implementation [18]. This expanded assay platform enables efficient functional assessment across a broader spectrum of pneumococcal serotypes relevant to next-generation vaccine evaluation.

## 2. Materials and Methods

### 2.1. Preparation of Pooled Sera for the Validation of Sixplexed Opsonophagocytic Killing Assay

The three pooled sera were collected from individuals previously vaccinated with pneumococcal conjugate or polysaccharide vaccines. Specifically, pooled sera were derived from donors who had received PCV13 (Pfizer, New York, NY, USA) followed by PPSV23 (Merck Sharp & Dohme; MSD, Kenilworth, NJ, USA), PCV20 (Pfizer), or PCV21 (MSD). Serum samples were collected at defined intervals after vaccination (26.4 ± 21.3 months for the PCV13/PPSV23 group, 15.2 ± 8.0 months for the PCV20 group, and 12.3 ± 1.4 months for the PCV21 group) and subsequently pooled to generate serum panels used for the optimization and validation of the sixplexed OPA. All participants provided written informed consent prior to sample collection, and the study protocol was approved by the Institutional Review Board of Korea University Guro Hospital (IRB No. 2023GR0518).

### 2.2. Target Bacterial Strains for Sixplexed Opsonophagocytic Killing Assay

Except for the 16 antibiotic-resistant serotypes provided by Professor Nahm at the University of Alabama at Birmingham, all pneumococcal serotypes examined in this study were isolated from patients with pneumococcal infection. Additional serotype confirmation was performed using a multiplex bead-based assay with monoclonal antibodies specific to eight serotypes (2, 4, 8, 11A, 12F, 17F, 20, and 22F) as described previously. To induce resistance to ciprofloxacin or doxycycline (Sigma-Aldrich, St. Louis, MO, USA), the selected pneumococcal serotype strains were subjected to stepwise exposure to increasing antibiotic concentrations, starting at 1000-fold below the target concentrations (16 µg/mL for ciprofloxacin and 2 µg/mL for doxycycline), with the antibiotic concentration doubled at each passage until the target concentration was reached. All antibiotics used in this study were purchased from Sigma-Aldrich.

### 2.3. Sixplexed Opsonophagocytic Killing Assay

The sixplexed OPA was performed as previously described, with minor modifications [19,20]. Briefly, HL-60 cells (ATCC, Manassas, VA, USA) were differentiated into granulocytes by culturing in RPMI 1640 medium (Corning Inc., Corning, NY, USA) supplemented with 10% fetal calf serum (FBS; Gibco, Grand Island, NY, USA) and 0.8% dimethylformamide (Fisher Scientific, Pittsburgh, PA, USA) for 5–6 days. Differentiated HL-60 cells were diluted to a final concentration of 5 × 10^6^ cells/mL in opsonization assay buffer B (OBB). Test serum samples (30 µL) were serially diluted in threefold increments with OBB in 96-well plates (SPL Life Sciences, Pocheon, Korea). Frozen working stocks of the target pneumococcal strains were thawed immediately prior to use, washed twice with OBB, and resuspended to a final concentration of 1 × 10^5^ CFU/mL in OBB. The six pneumococcal target strains were combined in equal proportions, and the resulting mixture was added to each well. After a 30-min incubation at room temperature, differentiated HL-60 cells and baby rabbit complement (BRC; Pel-Freez Biologicals, Rogers, AR, USA) were added to each well, followed by incubation at 37 °C for 45 min with shaking at 700 rpm. Effector-to-target (E:T) ratios ranging from 50:1 to 400:1 and complement concentrations between 7.5% and 17.5% were evaluated to determine optimal assay conditions. Following incubation, reaction mixtures were plated onto Todd Hewitt broth (Becton Dickinson, Franklin Lakes, NJ, USA) supplemented with 0.5% yeast extract (Becton Dickinson) and 1.5% Bacto agar (Becton Dickinson) (THYA). Following plating, 25 mL of Todd Hewitt yeast extract broth supplemented with 0.75% Bacto agar, one of six selective antibiotics, and 2,3,5-triphenyltetrazolium chloride (TTC; Sigma-Aldrich) was overlaid onto the spotted plates, which were then incubated overnight at 37 °C. Surviving bacterial colonies were enumerated, and opsonic indices (OIs) for each serum sample were calculated. The OI was defined as the highest serum dilution resulting in a 50% reduction in colony-forming units (CFUs) compared with the negative control, which contained heat-inactivated complement in place of diluted serum.

### 2.4. Validation of Sixplexed Opsonophagocytic Killing Assay

The sixplexed OPA was evaluated for specificity, accuracy, and precision using pooled serum samples. Assay specificity was assessed by inhibition OPA following pre-adsorption of serum samples with either homologous or heterologous serotype-specific polysaccharides (ATCC). Briefly, serum samples were mixed at a 1:1 ratio with polysaccharide at a final concentration of 20 µg/mL and incubated for 2 h at room temperature with gentle shaking. The opsonophagocytic killing assay was then performed as described above. Assay accuracy was evaluated by comparison with the single-serotype OPA (SOPA), using an E:T ratio of 200:1 for the multiplex OPA (MOPA) and 100:1 for SOPA. Assay precision was determined by conducting five independent experiments, with the coefficient of variation (CV) used as the measure of reproducibility.

### 2.5. Statistical Analysis

OPA titers were calculated as the mean of results obtained from two independent assays. Prior to statistical analysis, OPA titers were log_10_-transformed to approximate a normal distribution. For each serotype, geometric mean titers (GMTs) and corresponding 95% confidence intervals (CIs) were calculated.

Assay specificity was evaluated using inhibition OPA by calculating the percentage reduction in titers relative to the non-adsorbed control. Absolute serotype-specific GMTs and the degree of titer reduction following homologous and heterologous adsorption were compared using one-way analysis of variance (ANOVA), with the Kruskal–Wallis test applied for nonparametric data, followed by Tukey’s post hoc multiple-comparison test. Assay accuracy was assessed by comparing sixplexed OPA with SOPA using a paired *t*-test. Statistical significance was defined as a two-sided *p*-value < 0.05. Assay precision was considered acceptable when the CV was ≤30%. All statistical analyses were performed using GraphPad Prism (version 9.5) and SPSS (version 28).

## 3. Results

### 3.1. Development of Target Bacterial Strains for Sixplexed Opsonophagocytic Killing Assay

Eight additional pneumococcal serotypes (2, 4, 8, 11A, 12F, 17F, 20, and 22F) distinct from those included in the conventional fourplexed OPA were selected for this study. Candidate strains were initially screened from our clinical isolates to exclude pre-existing antibiotic resistance. A total of 12 antibiotics were subsequently evaluated, excluding optochin, spectinomycin, streptomycin, and trimethoprim. Among the remaining agents, penicillin G, amikacin, gentamicin, and kanamycin demonstrated cross-growth across multiple strains, whereas erythromycin, clindamycin, amoxicillin, nalidixic acid, and pyrimethamine failed to induce stable antibiotic resistance. Rifampicin was also excluded because agar discoloration interfered with colony visualization and enumeration. Based on these evaluations, ciprofloxacin and doxycycline were selected for resistance induction. As summarized in Table 1, ciprofloxacin (16 µg/mL) successfully induced resistance in serotypes 2, 4, 8, and 11A, while doxycycline (2 µg/mL) induced resistance in serotypes 12F, 17F, 20, and 22F, without cross-growth on agar plates containing other selective antibiotics.

### 3.2. Optimization of Sixplexed Opsonophagocytic Killing Assay

**Figure 1:**
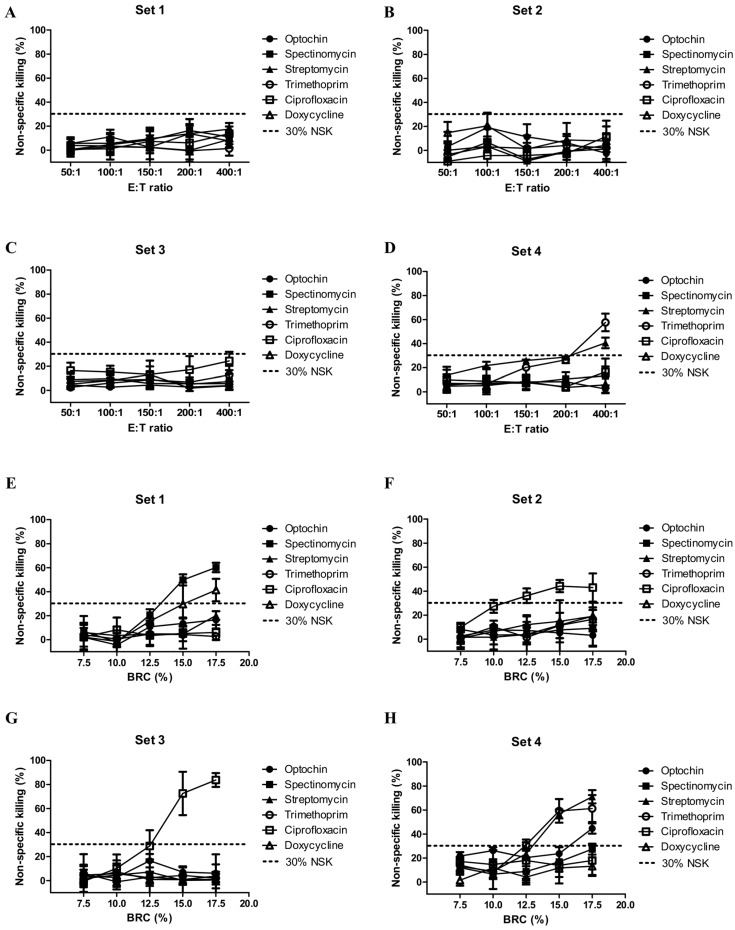
Evaluation of non-specific killing (NSK, %) across different effector-to-target (E:T) ratios (**A**–**D**) and baby rabbit complement (BRC) concentrations (**E**–**H**) for optimization of the sixplexed opsonophagocytic killing assay.

The sixplexed OPA was adapted from a previously described pneumococcal MOPA protocol with minor modifications to accommodate six serotypes per assay set. To determine the optimal E:T ratio for the sixplexed OPA, non-specific killing (NSK, %) was assessed and compared across a range of E:T ratios. As shown in Figure 1A–D, NSK remained below 30% for sets 1, 2, and 3 across all tested conditions. For set 4, which included serotypes 15B and 33F known to exhibit higher baseline antibody levels, NSK values below 30% were observed at E:T ratios ranging from 50:1 to 200:1. Based on these findings and to ensure consistency with the established fourplexed OPA conditions, an E:T ratio of 200:1 was selected for subsequent analyses.

After fixing the E:T ratio at 200:1, the concentration of BRC was incrementally increased in 2.5% steps and evaluated across a range of 7.5% to 17.5% (Figure 1E–H). Set 1 consistently maintained NSK levels below 30% under all tested conditions, even with simultaneous assessment of six serotypes. In contrast, for sets 2, 3, and 4, NSK values approached or slightly exceeded the 30% threshold at BRC concentrations of 12.5% or higher. To ensure robust assay performance under conditions of NSK consistently below 30% across all sets, the BRC concentration was optimized to 10% and used for subsequent validation experiments. Previous studies have reported that the optimal concentration of BRC in pneumococcal opsonophagocytic assays typically ranges from 8% to 16%, depending on the serotype and assay conditions [21]. In our optimization experiments, higher BRC concentrations resulted in increased NSK in several assay sets. Therefore, a concentration of 10% BRC was selected for the sixplexed OPA to maintain NSK below the recommended threshold while preserving adequate complement-mediated opsonophagocytic activity.

### 3.3. Specificity of Sixplexed Opsonophagocytic Killing Assay

**Figure 2:**
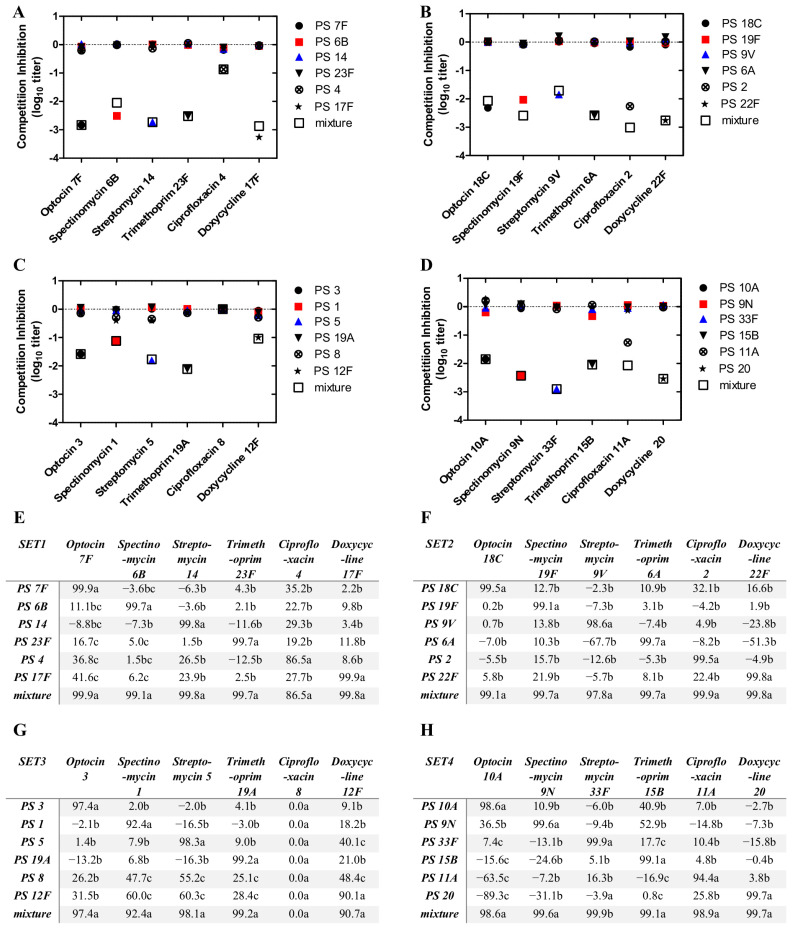
Specificity analysis of the sixplexed opsonophagocytic killing assay across assay (OPA) sets. (**A**–**D**) Inhibition OPA titers are presented as dot plots following log_10_ transformation. (**E**–**H**) Percent reduction in OPA titers relative to the non-adsorbed control is shown. Values marked with different superscript letters within the same column indicate statistically significant differences (*p* < 0.05).

Following pre-adsorption of pooled sera with homologous or heterologous capsular polysaccharides, assay specificity for serotypes included in each set was evaluated using inhibition OPA (Figure 2; Appendix A Table A3, Table A4, Table A5 and Table A6). In set 1, five serotypes (7F, 6B, 14, 23F, and 17F) exhibited near-complete inhibition following homologous polysaccharide adsorption, whereas heterologous adsorption resulted in less than a 50% reduction in OPA titers, confirming serotype-specific responses. In contrast, serotype 4 demonstrated markedly low baseline OPA titers (30 ± 3.1). Upon homologous polysaccharide adsorption, titers decreased to the assay detection limit (OI = 4), while heterologous adsorption resulted in a reduction to 21.7 ± 2.1% (26.8 ± 5.5%) of the non-adsorbed control. Consistent with these findings, polysaccharide-specific IgG concentrations for serotype 4 were low, measuring 2.7 ± 0.05, 3.3 ± 0.5, and 0.6 ± 0.00 µg/mL in pooled sera 1, 2, and 3, respectively (Appendix A Table A2). Pneumococcal ELISA was performed to measure the polysaccharide serotype-specific IgG titers as previously described (http://www.vaccine.uab.edu (20 March 2026)). To exclude strain-related factors, nanopore sequencing was performed and confirmed the absence of genetic mutations affecting serotype identity.

In sets 2 and 4, homologous polysaccharide adsorption resulted in reductions of ≥90% across all included serotypes, whereas heterologous adsorption consistently produced reductions of <50%, indicating high serotype specificity. Similarly, in set 3, homologous polysaccharide adsorption led to >90% inhibition for all serotypes except serotype 8, which exhibited an opsonic index at the assay detection limit (OI = 4 ± 0.0). Despite the absence of detectable OPA activity, polysaccharide-specific IgG concentrations for serotype 8 were measurable, with levels of 12.6 ± 0.4, 6.3 ± 0.1, and 5.7 ± 0.1 µg/mL in pooled sera 1, 2, and 3, respectively. To exclude serotype misclassification or strain-related artifacts, nanopore sequencing of the ciprofloxacin-resistant serotype 8 isolate included in set 3 was performed and confirmed its identity as serotype 8.

Collectively, these inhibition OPA results demonstrated high assay specificity across all 24 serotypes evaluated in the four sixplexed assay sets.

### 3.4. Accuracy of Sixplexed Opsonophagocytic Killing Assay

**Figure 3:**
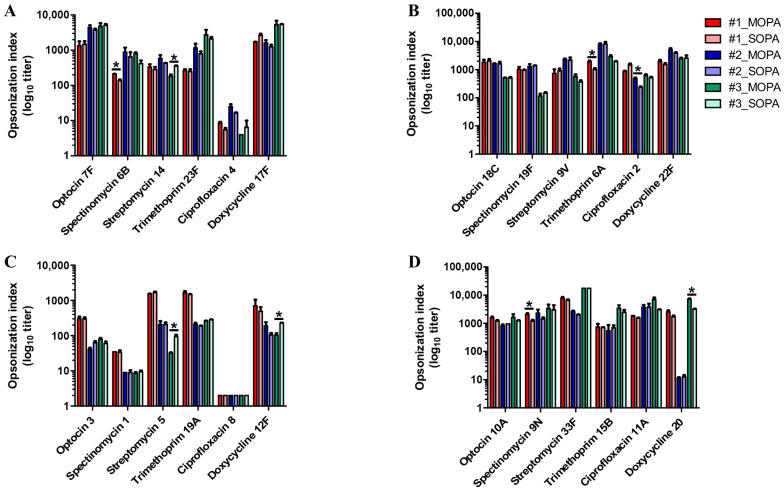
Comparison of opsonophagocytic killing assay (OPA) titers obtained using the sixplexed OPA and the single-serotype OPA (SOPA). The y-axis is presented on a log_10_ scale for better visualization of the wide range of data points. (**A**) OPA titers for set 1, (**B**) OPA titers for set 2, (**C**) OPA titers for set 3, (**D**) OPA titers for set 4. Asterisks (*) indicate a significant difference (*p* < 0.05) between SOPA and MOPA.

Assay accuracy was further evaluated by comparing sixplexed OPA results with those obtained using the previously described SOPA (Figure 3; Appendix A Table A7). Sixplexed OPA and SOPA were performed in parallel using three pooled serum samples.

In set 1, sixplexed OPA titers were modestly higher than SOPA titers for serotype 6B in pooled serum 1 (1.55-fold) and for serotype 14 in pooled serum 3 (0.51-fold difference). For the remaining serotypes (7F, 23F, 4, and 17F), no significant differences were observed between sixplexed OPA and SOPA across the three pooled sera. Similarly, no significant differences were detected for serotypes 6B and 14 in the other pooled sera. Overall, fold differences between sixplexed OPA and SOPA ranged from 0.91 to 1.17 for 7F, 1.36 to 1.91 for 6B, 0.51 to 1.34 for 14, 1.07 to 1.45 for 23F, 0.62 to 1.55 for 4, and 0.65 to 1.27 for 17F, indicating strong concordance between the two assays. In set 2, statistically significant differences between sixplexed OPA and SOPA were observed under only two conditions, while no significant differences were detected across the remaining pooled sera. Specifically, sixplexed OPA titers were higher for serotype 6A in pooled serum 1 (1.90-fold) and for serotype 2 in pooled serum 2 (2.01-fold). For the other serotypes in set 2, the fold differences between sixplexed OPA and SOPA remained within a comparable range (0.89–1.01 for 18C, 0.79–1.06 for 19F, 0.80–1.57 for 9V, 0.95–1.90 for 6A, 0.60–2.01 for 2, and 0.95–1.30 for 22F), indicating overall concordance between the two assays. Similarly, in set 4, sixplexed OPA titers were modestly higher for serotype 9N in pooled serum 1 (1.70-fold) and for serotype 20 in pooled serum 3 (2.25-fold) compared with SOPA. No significant differences were observed for the remaining serotypes across pooled sera. Overall fold differences ranged from 0.85 to 1.31 for 10A, 1.12 to 1.70 for 9N, 1.00 to 1.27 for 33F, 0.78 to 1.40 for 15B, 0.98 to 2.28 for 11A, and 0.92 to 2.25 for 20, further supporting strong agreement between sixplexed OPA and SOPA.

In contrast, in set 3, SOPA yielded significantly higher OPA titers than sixplexed OPA for serotypes 5 (2.97-fold) and 12F (2.24-fold), observed only in pooled serum 3. No significant differences were detected for the remaining pooled sera. Across all serotypes in set 3, fold differences between sixplexed OPA and SOPA ranged from 0.65 to 1.31 for serotype 3, 0.89 to 1.03 for serotype 1, 0.34 to 1.00 for serotype 5, 0.92 to 1.12 for serotype 19A, 1.00 for serotype 8, and 0.45 to 1.81 for serotype 12F.

Overall, the sixplexed OPA demonstrated strong concordance with SOPA, achieving an accuracy of 91.67% (66 of 72 tested conditions).

### 3.5. Precision of Sixplexed Opsonophagocytic Killing Assay

The precision of the sixplexed OPA under standard operating conditions was evaluated across five independent experiments using three pooled serum samples. For each serotype, mean OPA titers, standard deviations, and CVs were calculated.

Across all assay sets, the sixplexed OPA demonstrated acceptable precision, with CVs below 30% for the majority of serotypes evaluated (Table 2). In set 1, CVs for five serotypes ranged from 4.2% to 25.4%, while in sets 2 and 4, all 12 serotypes exhibited CVs below 30% across the three pooled sera (2.9–19.7% for set 2 and 2.5–24.3% for set 4). Similarly, in set 3, five serotypes showed CVs ranging from 5.2% to 19.5%. In contrast, elevated variability was observed for serotypes 4 (set 1) and 8 (set 3), with CVs exceeding 30% (up to 44.2% and 69.0%, respectively); notably, all such cases were associated with very low OPA titers (<50), consistent with increased assay variability near the lower limit of detection.

## 4. Discussion

In this study, we successfully developed and validated a sixplexed OPA that expands functional antibody assessment from 16 to 24 pneumococcal serotypes within four assay sets. Against the backdrop of serotype replacement and the increasing contribution of non-vaccine and antimicrobial-resistant serotypes to IPDs, this methodological advance addresses a critical gap in immunogenicity evaluation for next-generation high-valency PCVs. The sixplexed OPA demonstrated high serotype specificity across all 24 serotypes, strong concordance with the established SOPA, and acceptable precision under standardized assay conditions, with observed variability largely confined to serotypes exhibiting very low opsonic activity near the assay detection limit. Importantly, optimization of E:T ratios and complement concentrations ensured non-specific killing remained below predefined thresholds, enabling reliable multiplexed measurement without compromising assay performance. Collectively, these findings support the sixplexed OPA as a robust, scalable, and high-throughput platform for functional immune evaluation, with direct applicability to vaccine development, comparative immunogenicity studies, and surveillance of emerging pneumococcal serotypes in the post-PCV era.

The serotype-specific heterogeneity observed in opsonophagocytic activity highlights important biological and immunological differences that extend beyond assay performance. In this study, certain serotypes, notably serotypes 4 and 8, consistently exhibited very low OPA titers and increased variability despite measurable polysaccharide-specific IgG concentrations. This discordance suggests that quantitative antibody levels alone may not adequately reflect functional opsonic capacity, reinforcing the importance of OPA as a functional correlate of protection. Differences in capsular polysaccharide structure, epitope accessibility, antibody avidity, and Fc-mediated effector functions may all contribute to reduced opsonophagocytic efficiency in these serotypes [22,23]. Low OPA titers were also associated with increased CV, particularly near the lower limit of assay detection. Such variability is a well-recognized characteristic of functional assays when opsonic activity approaches background levels and should not be interpreted as assay instability [24]. Rather, these findings likely reflect intrinsic limitations in functional antibody responses against specific serotypes. Importantly, nanopore sequencing confirmed the serotype identity of isolates with low OPA titers, thereby excluding strain misclassification or genetic alterations as contributing factors. Additionally, previous studies investigating ciprofloxacin-resistant *S. pneumoniae* have shown that low-level resistance isolates (2 µg/mL) typically exhibit no detectable genetic alterations, whereas high-level resistance isolates (128 µg/mL) harbor mutations in the quinolone resistance-determining regions (QRDRs) of the *parC* and *gyrA* genes [25]. Although a concentration of 16 µg/mL was used to induce resistance in the present study, fluoroquinolone resistance in pneumococci is well established to arise primarily from mutations in these loci. The *parC* and *gyrA* genes encode subunits of type IV topoisomerase and DNA gyrase, respectively, enzymes essential for DNA replication and chromosome maintenance rather than determinants of capsule expression or known virulence factors [26]. Importantly, there is currently no evidence linking these mutations to altered surface antigenicity or immune evasion. Accordingly, while induction of ciprofloxacin resistance in serotypes 4 and 8 may influence bacterial growth characteristics, it is unlikely to substantially affect opsonophagocytic susceptibility. In our assay, E:T ratios were standardized during interaction with differentiated HL-60 cells to ensure consistent phagocytic conditions. Taken together, these genetic and experimental considerations suggest that the antibiotic-resistant strains used in this study remain appropriate for functional assessment in the multiplex OPA platform.

In the context of World Health Organization (WHO) guidance on pneumococcal OPAs, the sixplexed OPA developed in this study aligns well with key performance criteria, including serotype specificity, assay reproducibility, and concordance with established single-serotype OPAs [27]. The assay demonstrated acceptable precision across most serotypes, with CV generally below the WHO-recommended threshold [27], and showed strong agreement with SOPA results, supporting its validity as a functional measure of vaccine-induced immunity. Importantly, optimization of E:T ratios and complement concentrations ensured controlled NSK, consistent with WHO recommendations for assay robustness and standardization [20,28].

However, several limitations should be considered when interpreting these findings in the context of WHO standards. For serotypes exhibiting very low opsonic activity, increased variability was observed near the lower limit of assay detection, which may constrain quantitative interpretation. This phenomenon is recognized within WHO OPA guidance as an inherent characteristic of functional assays rather than a methodological shortcoming; nevertheless, it underscores the need for cautious interpretation of low OPA titers and highlights the potential value of complementary immunological endpoints. Additionally, although the sixplexed format substantially improves analytical throughput and scalability, further inter-laboratory validation will be essential to support broader implementation, standardization, and potential regulatory acceptance in alignment with WHO recommendations.

In summary, the sixplexed OPA offers a scalable and efficient platform for functional antibody assessment across an expanded range of pneumococcal serotypes. This platform is well suited to support immunogenicity evaluation and comparative studies of current and future high-valency pneumococcal vaccines.

## Figures and Tables

**Table 1:** Composition of target bacterial strains and antibiotic resistance profiles of assay sets used in the sixplexed opsonophagocytic killing assay.

Resistant Antibiotic		Optocin (8 μg/mL)	Spectinomycin (300 μg/mL)	Streptomycin (300 μg/mL)	Trimethoprim (25 μg/mL)	Ciprofloxacin (8 μg/mL)	Doxycycline (2 μg/mL)
**SET1**	**Serotype**	**7F**	**6B**	**14**	**23F**	**4**	**17F**
	Origin	DS2617-97	BG25-9	DS2214-94	1212458	TIGR4	S8576
	Source (year)	BEI Resources	BEI Resources	BEI Resources	BEI Resources	ATCC ^1^	KUGH ^2^ (2011)
**SET2**	**Serotype**	**18C**	**19F**	**9V**	**6A**	**2**	**22F**
	Origin	GP116	2217-94	1081748	EF6796	D39	SP19-86
	Source (year)	BEI Resources	BEI Resources	BEI Resources	BEI Resources	BEI Resources	KUGH ^2^ (2019)
**SET3**	**Serotype**	**3**	**1**	**5**	**19A**	**8**	**12F**
	Origin	Wu2	L82006	DBL5	DS3519-97	MNX2191	DS001
	Source (year)	BEI Resources	BEI Resources	BEI Resources	BEI Resources	BEI Resources	HUDSH ^3^ (2018)
**SET4**	**Serotype**	**10A**	**9N**	**33F**	**15B**	**11A**	**ST20**
	Origin	DS3032-06	DS1398-00	DS3052-06	DS0556-97	MNX1951	SP19-101
	Source (year)	BEI Resources	BEI Resources	BEI Resources	BEI Resources	BEI Resources	KUGH ^2^ (2019)

^1^ ATCC (American Type Culture Collection); ^2^ KUGH (Korea University Guro hospital); ^3^ HUDSH (Hallym University Dongtan Sacred Heart Hospital).

**Table 2:** Intra-assay precision of the sixplexed opsonophagocytic killing assay.

	SET1			SET2			SET3			SET4		
	Serotype	Average ± STD	CV (%)	Serotype	Average ± STD	CV (%)	Serotype	Average ± STD	CV (%)	Serotype	Average ± STD	CV (%)
**sera #1**	**7F**	1977 ±95	4.8	**18C**	6659 ±1311.1	19.7	**3**	284 ±48.1	17.0	**10A**	361 ±76.6	21.2
	**6B**	727 ±33.8	4.6	**19F**	3002 ±500.2	16.7	**1**	52 ±2.7	5.2	**9N**	1150 ±221.2	19.2
	**14**	1976 ±230.2	11.6	**9V**	2341 ±239.4	10.2	**5**	656 ±128.1	18.7	**33F**	4716 ±572.1	12.1
	**23F**	886 ±80.8	9.1	**6A**	4729 ±513.6	10.9	**19A**	1067 ±75.8	7.1	**15B**	501 ±71.5	14.3
	**4**	10 ±1.2	12.2	**2**	6895 ±198.5	2.9	**8**	38 ±17.1	45.5	**11A**	2036 ±119.0	5.8
	**17F**	2795 ±527.5	18.9	**22F**	4744 ±650.2	13.7	**12F**	539 ±105.0	19.5	**20**	1662 ±136.5	8.2
**sera #2**	**7F**	2,154,347.6	16.1	**18C**	2540 ±182.2	7.2	**3**	25 ±3.5	14.1	**10A**	2461 ±427.0	17.3
	**6B**	587 ±67.2	11.5	**19F**	1894 ±101.7	5.4	**1**	48 ±2.6	5.4	**9N**	2831 ±462.0	16.3
	**14**	451 ±60.5	13.4	**9V**	2608 ±332.0	12.7	**5**	432 ±57.3	13.2	**33F**	7946 ±198.6	2.5
	**23F**	829 ±97.5	11.8	**6A**	9318 ±1476.2	15.8	**19A**	908 ±165.3	18.2	**15B**	1436 ±227.6	15.9
	**4**	38 ±9.8	25.4	**2**	803 ±122.6	15.3	**8**	4 ±0.9	24.8	**11A**	4505 ±690.1	15.3
	**17F**	2285 ±359.5	15.7	**22F**	4489 ±649.6	14.5	**12F**	1334 ±111.1	8.3	**20**	5455 ±784.9	14.4
**sera #3**	**7F**	2688 ±519.5	19.3	**18C**	2732 ±367.3	13.4	**3**	41 ±11.4	27.9	**10A**	2869 ±697.5	24.3
	**6B**	95 ±4.0	4.2	**19F**	437 ±83.3	19.1	**1**	10 ±1.3	13.3	**9N**	2291 ±210.2	9.2
	**14**	149 ±15.0	10.0	**9V**	3287 ±590.6	18.0	**5**	243 ±47.5	19.5	**33F**	2889 ±324.4	11.2
	**23F**	1188 ±109.8	9.2	**6A**	19,506 ±2658.2	13.6	**19A**	806 ±145.3	18.0	**15B**	2660 ±514.7	19.4
	**4**	8 ±3.4	44.2	**2**	5866 ±367.7	6.3	**8**	8 ±5.4	69.0	**11A**	469 ±97.4	20.7
	**17F**	4697 ±677.6	14.4	**22F**	13,516 ±1328.4	9.8	**12F**	979 ±112.1	11.5	**20**	1765 ±248.4	14.1

STD, standard deviation; CV, coefficient of variation.

## Data Availability

The data presented in this study are available in the article and Appendix A.

## Abbreviations

The following abbreviations are used in this manuscript:

PCVs	Pneumococcal conjugate vaccines
PPSVs	Pneumococcal polysaccharide vaccines
IPD	Invasive pneumococcal disease
E:T ratio	Effector-to-target ratio
BRC	Baby rabbit complement
NSK	Non-specific killing
OPA	Opsonophagocytic killing assay
SOPA	Single-serotype OPA
CV	Coefficient of variation
CFUs	Colony-forming units
GMTs	Geometric mean titers
CIs	Confidence intervals
OIs	Opsonic indices

## Appendix A

**Table A1:** Serotype composition of pneumococcal vaccines.

Vaccine	Serotypes																															
	1	2	3	4	5	6A	6B	7F	8	9N	9V	10A	11A	12F	14	15A	15B	15C	16F	17F	18C	19A	19F	20	22F	23A	23B	23F	24F	31	33F	35B
**PPSV23**																																
**PCV7**																																
**PCV10**																																
**PCV13**																																
**PCV15**																																
**PCV20**																																
**PCV21**																																
**PCV21 ***																																
**PCV24 ***																																

Colors represent the manufacturers: Green (Merck Sharp & Dohme, MSD), Gray (GlaxoSmithKline, GSK), BLUE (Pfizer), YELLOW (SK bioscience and Sanofi pasteur). * Characters indicate vaccines that are undergoing clinical trials.

**Table A2:** Geometric mean titers (µg/mL) with standard deviations of capsular polysaccharide-specific IgG.

**SET1**	**Sera #1**	**Sera #2**	**Sera #3**	**SET2**	**Sera #1**	**Sera #2**	**Sera #3**
**7F**	9.45 ± 0.17	6.45 ± 0.07	8.83 ± 0.03	**18C**	6.93 ± 0.69	5.44 ± 0.68	5.96 ± 0.98
**6B**	8.71 ± 0.03	9.41 ± 0.11	4.31 ± 0.01	**19F**	14.49 ± 0.09	8.23 ± 0.11	2.52 ± 0.01
**14**	26.75 ± 3.61	15.14 ± 2.29	19.57 ± 0.73	**9V**	4.57 ± 0.01	7.61 ± 0.29	3.45 ± 0.20
**23F**	4.24 ± 0.23	6.16 ± 0.46	6.30 ± 0.69	**6A**	4.43 ± 0.17	6.72 ± 0.56	5.15 ± 0.40
**4**	2.7 ± 0.05	3.3 ± 0.5	0.6 ± 0.00	**2**	12.23 ± 0.01	2.01 ± 0.03	4.62 ± 0.03
**17F**	10.99 ± 0.1	9.94 ± 0.45	8.85 ± 0.13	**22F**	5.22 ± 0.16	4.18 ± 0.20	2.78 ± 0.33
**SET3**	**sera #1**	**sera #2**	**sera #3**	**SET4**	**sera #1**	**sera #2**	**sera #3**
**3**	1.24 ± 0.04	0.73 ± 0.05	0.64 ± 0.07	**10A**	9.76 ± 0.26	12.73 ± 0.38	12.29 ± 0.43
**1**	6.60 ± 0.19	6.26 ± 0.35	1.84 ± 0.10	**9N**	4.56 ± 0.29	3.60 ± 0.48	8.43 ± 0.81
**5**	5.98 ± 0.33	3.46 ± 0.15	1.17 ± 0.14	**33F**	11.17 ± 0.55	9.29 ± 0.73	9.42 ± 0.94
**19A**	10.39 ± 1.05	8.31 ± 1.18	6.65 ± 1.09	**15B**	12.35 ± 0.23	16.77 ± 0.51	16.00 ± 0.17
**8**	12.6 ± 0.4	6.3 ± 0.1	5.7 ± 0.1	**11A**	5.54 ± 0.17	7.07 ± 0.15	5.99 ± 0.02
**12F**	3.86 ± 0.25	4.40 ± 1.22	3.77 ± 0.51	**20**	14.18 ± 0.10	5.59 ± 0.09	12.85 ± 0.21

**Table A3:** Geometric mean titers with standard deviations for the specificity analysis of set 1 in sixplexed opsonophagocytic killing assay.

Serotype	Capsular Polysaccharide as an Inhibitor (100 μg/mL)							
	None	PS 7F	PS 6B	PS 14	PS 23F	PS 4	PS 17F	Mixture
**Optochin** **7F**	2725 ±233.9	4 ±0.0	2422 ±694.4	2965 ±732.1	2269 ±501.4	1721 ±30.1	1592 ±75.7	4 ±0.0
**Spectinomycin 6B**	1281 ±20.3	1327 ±21.2	4 ±0.0	1375 ±43.9	1218 ±36.5	1262 ±80.0	1202 ±13.0	12 ±1.1
**Streptomycin** **14**	2130 ±251.8	2265 ±461.2	2207 ±387.4	4 ±0.0	2099 ±433.8	1565 ±13.4	1621 ±64.9	4 ±0.0
**Trimethoprim 23F**	1339 ±110.1	1282 ±104.6	1311 ±34.2	1494 ±264.3	4 ±0.0	1506 ±57.7	1306 ±66.4	4 ±0.0
**Ciprofloxacin** **4**	30 ±3.1	19 ±0.2	23 ±3.0	21 ±0.9	24 ±2.8	4 ±0.0	21 ±0.0	4 ±0.0
**Doxycycline 17F**	7344 ±472.8	7183 ±528.8	6624 ±273.7	7097 ±459.7	6478 ±227.8	6710 ±202.5	4 ±0.0	14 ±10.1

**Table A4:** Geometric mean titers with standard deviations for the specificity analysis of set 2 in sixplexed opsonophagocytic killing assay.

Serotype	Capsular Polysaccharide as an Inhibitor (100 μg/mL)							
	None	PS 18C	PS 19F	PS 9V	PS 6A	PS 2	PS 22F	Mixture
**Optochin** **18C**	1352 ±21.9	7 ±3.3	1350 ±50.1	1343 ±63.3	1447 ±33.4	1426 ±124.5	1274 ±136.1	12 ±0.1
**Spectinomycin 19F**	1563 ±124.4	1364 ±90.1	15 ±1.7	1347 ±37.6	1402 ±8.4	1318 ±114.2	1220 ±16.0	4 ±0.0
**Streptomycin 9V**	1599 ±19.2	1636 ±143.7	1716 ±197.8	23 ±3.5	2681 ±680.7	1800 ±182.2	1689 ±51.0	35 ±17.1
**Trimethoprim 6A**	1561 ±131.8	1351 ±51.3	1790 ±93.0	1628 ±114.3	4 ±0.0	1596 ±82.6	1393 ±32.0	4 ±0.0
**Ciprofloxacin** **2**	4057 ±257.9	2755 ±203.2	4227 ±567.4	3857 ±220.8	4389 ±322.3	22 ±0.9	3149 ±313.4	4 ±0.0
**Doxycycline 22F**	2285 ±381.0	1905 ±385.7	2242 ±453.9	2829 ±97.0	3458 ±342.5	2396 ±360.2	4 ±0.0	4 ±0.0

**Table A5:** Geometric mean titers with standard deviations for the specificity analysis of set 3 in sixplexed opsonophagocytic killing assay.

Serotype	Capsular Polysaccharide as an Inhibitor (100 μg/mL)							
	None	PS 3	PS 1	PS 5	PS 19A	PS 8	PS 12F	Mixture
**Optochin** **3**	151 ±17.3	4 ±0.0	155 ±23.6	149 ±16.1	171 ±9.0	112 ±15.7	104 ±10.7	4 ±0.0
**Spectinomycin** **1**	53 ±0.8	52 ±2.3	4 ±0.0	49 ±3.8	49 ±2.1	28 ±6.7	21 ±0.6	4 ±0.0
**Streptomycin** **5**	395 ±2.5	403 ±7.3	460 ±23.7	7 ±2.7	459 ±24.8	177 ±2.3	157 ±2.8	8 ±3.5
**Trimethoprim** **19A**	521 ±3.0	499 ±19.1	536 ±22.7	474 ±18.9	4 ±0.0	390 ±5.7	373 ±25.9	4 ±0.0
**Ciprofloxacin** **8**	4 ±0.0	4 ±0.0	4 ±0.0	4 ±0.0	4 ±0.0	4 ±0.0	4 ±0.0	4 ±0.0
**Doxycycline** **12F**	105 ±0.3	95 ±14.2	86 ±9.2	63 ±3.5	83 ±10.2	54 ±0.7	10 ±0.4	10 ±1.0

**Table A6:** Geometric mean titers with standard deviations for the specificity analysis of set 4 in sixplexed opsonophagocytic killing assay.

Serotype	Capsular Polysaccharide as an Inhibitor (100 μg/mL)							
	None	PS 10A	PS 9N	PS 33F	PS 15B	PS 11A	PS 20	Mixture
**Optochin** **10A**	285 ±2.8	4 ±0.0	181 ±6.2	264 ±48.0	330 ±30.2	466 ±58.3	540 ±108.0	4 ±0.0
**Spectinomycin** **9N**	1084 ±98.1	966 ±155.5	4 ±0.0	1226 ±100.0	1351 ±102.7	1162 ±139.6	1421 ±52.8	4 ±0.0
**Streptomycin** **33F**	3195 ±60.5	3386 ±120.2	3494 ±24.0	4 ±0.0	3032 ±225.1	2675 ±70.7	3320 ±426.3	4 ±0.0
**Trimethoprim** **15B**	441 ±49.5	261 ±1.3	208 ±6.0	363 ±53.5	4 ±0.0	515 ±75.5	437 ±1.0	4 ±0.0
**Ciprofloxacin** **11A**	1008 ±50.0	938 ±54.1	1158 ±107.7	904 ±22.7	960 ±31.6	57 ±10.2	748 ±32.8	11 ±7.4
**Doxycycline** **20**	1378 ±126.3	1414 ±389.7	1478 ±129.6	1595 ±95.0	1383 ±43.3	1325 ±39.0	4 ±0.0	4 ±0.0

**Table A7:** Comparison of geometric mean titers with standard deviations obtained from the sixplexed opsonophagocytic killing assay (M) versus those from the single opsonophagocytic killing assay (S).

		**SET1**						**SET2**					
		**7F**	**6B**	**14**	**23F**	**4**	**17F**	**18C**	**19F**	**9V**	**6A**	**2**	**22F**
*sera #1*	M	1356 ±300	213 ±10.5 *	335 ±45.5	269 ±15.5	9 ±0.5	1737 ±25.5	1836 ±299.5	1025 ±159.5	729 ±206.0	1928 ±112.5 *	882 ±22.0	1973 ±163.0
	S	1488 ±218.5	137 ±7.0	285 ±35.0	251 ±25.5	6 ±0.5	2671 ±191.5	2053 ±223.5	964 ±30.0	909 ±121.0	1015 ±82.0	1479 ±110.5	1526 ±124.5
*sera #2*	M	4404 ±487.0	879 ±217.0	583 ±103.	1163 ±264.5	25 ±3.0	1599 ±237.5	1639 ±25.0	1292 ±188.5	2321 ±109.5	7722 ±623.5	480 ±32.5 *	5071 ±505.0
	S	3750 ±251.5	649 ±161.5	434 ±10.0	800 ±93.0	17 ±0.5	1256 ±121.5	1622 ±194.0	1397 ±16.0	2180 ±368.0	8142 ±1004.5	238 ±11.0	3892 ±193.5
*sera #3*	M	4795 ±770.0	801 ±48.0	182 ±16.0 *	2754 ±738.5	4 ±0.0	5340 ± 1100.5	516.5 ±6.5	116 ±16.0	576 ±72.0	2877 ±253.0	627 ±40.0	2444.5 ±147.5
	S	5159 ±284.5	420 ±63.5	358 ±9.5	2118 ±177.5	7 ±2.5	5476 ±45.0	509 ±24.0	147 ±8.0	368 ±34.5	1940 ±45.5	519 ±24.0	2562 ±466.0
		**SET3**						**SET4**					
		**3**	**1**	**5**	**19A**	**8**	**12F**	**10A**	**9N**	**33F**	**15B**	**11A**	**20**
*sera #1*	M	308 ±28.0	35 ±0.0	1527 ±31.0	1679 ±117.5	2 ±0.0	705 ±245.5	1572 ±107.5	2055 ± 149.0 *	7837 ±547.0	736 ±155.0	1836 ±7.5	2556 ±239.5
	S	299 ±24.5	34 ±3.0	1694 ±64.0	1501 ±14.5	2 ±0.0	494 ±107.0	1215 ±71.0	1210 ±86.5	6678 ±265.0	724 ±5.0	1532 ±44.5	1712 ±111.5
*sera #2*	M	41 ±3.0	9 ±0.0	209 ±36.5	212 ±14.5	2 ±0.0	190 ±36.0	810 ±72.0	2325 ±513.0	2590 ±152.0	549 ±224.5	3694 ±542.0	12 ±0.5
	S	64 ±4.5	9 ±1.0	210 ±19.5	191 ±2.5	2 ±0.0	105 ±10.0	958 ±2.0	1440 ±130.5	2033 ±29.5	700 ±102.5	3764 ±843.0	13 ±1.5
*sera #3*	M	79 ±5.0	8.5 ±0.5	32 ±1.0 *	263 ±3.0	2 ±0.0	103.5 ±9.5 *	1624 ±333.0	3357 ±880.5	17,496 ±0.0	3445 ±649.5	7041 ±820.0	7194 ±268.0 *
	S	61 ±5.5	10 ±0.5	95 ±8.0	287 ±2.5	2 ±0.0	232 ±2.0	1236 ±41.5	3003 ±978.0	17,496 ±0.0	2469 ±350.0	3090 ±49.0	3195 ±76.0

* Values marked with different superscript letters within the same row for the sixplexed OPA (M) are significantly different (*p* < 0.05).
